# The oncogenic mechanisms of the Janus kinase-signal transducer and activator of transcription pathway in digestive tract tumors

**DOI:** 10.1186/s12964-023-01421-9

**Published:** 2024-01-25

**Authors:** Ruihong Zhao, Zhangmin Hu, Xiaoli Zhang, Shujuan Huang, Guodong Yu, Zhe Wu, Wei Yu, Juan Lu, Bing Ruan

**Affiliations:** State Key Laboratory for Diagnosis and Treatment of Infectious Diseases, National Clinical Research Center for Infectious Diseases, Collaborative Innovation Center for Diagnosis and Treatment of Infectious Diseases, The First Affiliated Hospital, National Medical Center for Infectious Diseases, Zhejiang University School of Medicine, No. 79 Qingchun Road, Shangcheng District, Hangzhou, Zhejiang 310003 China

**Keywords:** JAK, STAT, Digestive tract tumors, Biological functions, Oncogenic mechanism

## Abstract

Digestive tract tumors are heterogeneous and involve the dysregulation of multiple signaling pathways. The Janus kinase-signal transducer and activator of transcription (JAK–STAT) pathway plays a notable role in the oncogenesis of digestive tract tumors. Typically activated by pro-inflammatory cytokines, it regulates important biological processes, such as cell growth, differentiation, apoptosis, immune responses, and inflammation. The aberrant activation of this pathway manifests in different forms, including mutations in JAKs, overexpression of cytokine receptors, and sustained STAT activation, and contributes to promoting the malignant characteristics of cancer cells, including uncontrolled proliferation, resistance to apoptosis, enhanced invasion and metastasis, angiogenesis, acquisition of stem-like properties, and drug resistance. Numerous studies have shown that aberrant activation of the JAK-STAT pathway is closely related to the development and progression of digestive tract tumors, contributing to tumor survival, angiogenesis, changes in the tumor microenvironment, and even immune escape processes. In addition, this signaling pathway also affects the sensitivity of digestive tract tumors to chemotherapy and targeted therapy. Therefore, it is crucial to comprehensively understand the oncogenic mechanisms underlying the JAK-STAT pathway in order to develop effective therapeutic strategies against digestive tract tumors. Currently, several JAK–STAT inhibitors are undergoing clinical and preclinical trials as potential treatments for various human diseases. However, further investigation is required to determine the role of this pathway, as well as the effectiveness and safety of its inhibitors, especially in the context of digestive tract tumors. In this review, we provide an overview of the structure, classic activation, and negative regulation of the JAK-STAT pathway. Furthermore, we discuss the pathogenic mechanisms of JAK-STAT signaling in different digestive tract tumors, with the aim of identifying potential novel therapeutic targets.

Video Abstract

**Supplementary Information:**

The online version contains supplementary material available at 10.1186/s12964-023-01421-9.

## Introduction

**Fig. 1:**
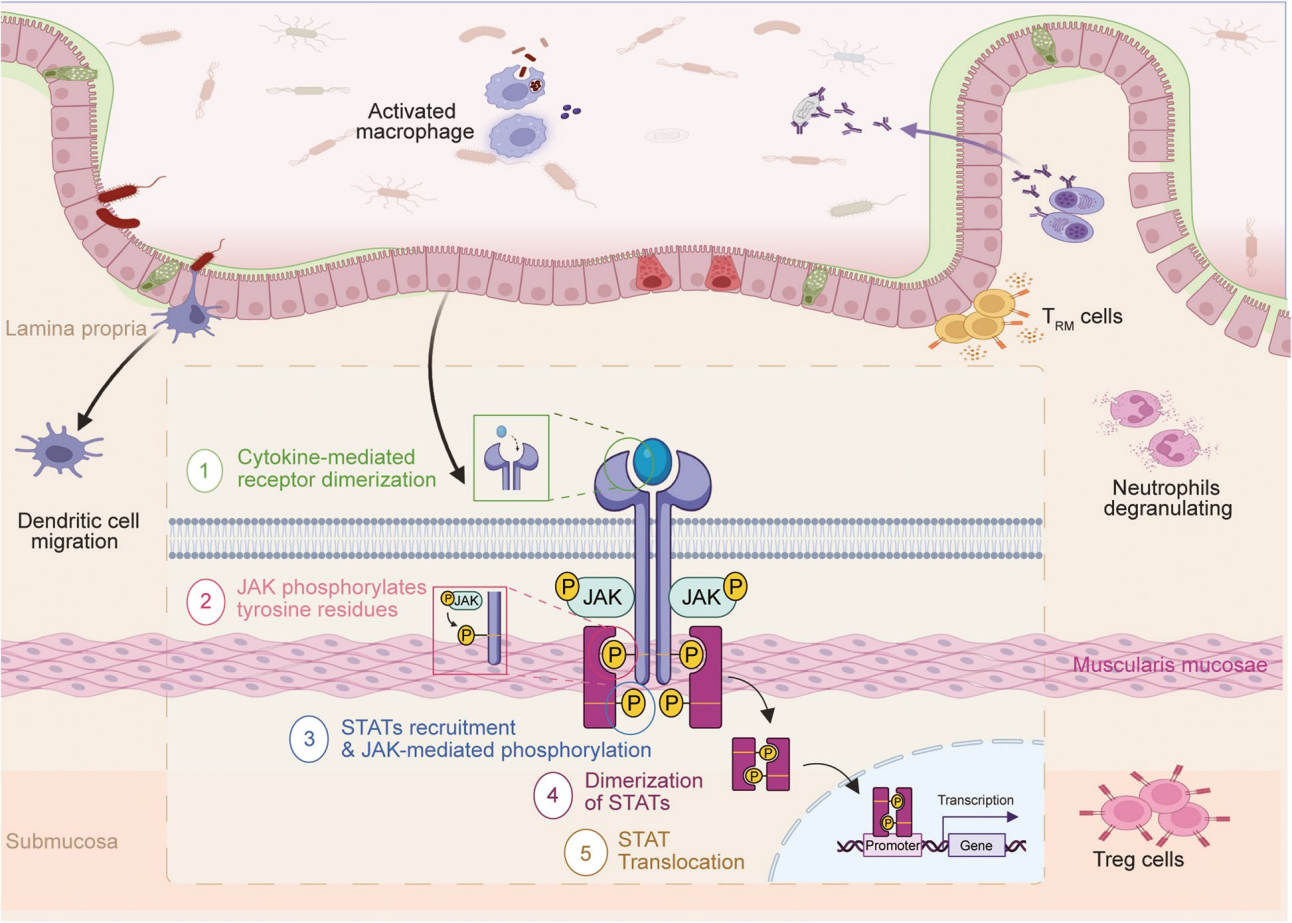
The components and activation of the Janus kinase-signal transducer and activator of transcription (JAK-STAT) pathway

The Janus kinase-signal transducer and activator of transcription (JAK-STAT) pathway is a crucial cell signaling pathway that is frequently activated by an extensive repertoire of extracellular cytokines and growth factors [1–3]. It plays a critical role in regulating essential biological processes, including cellular processes, inflammation, and immunological responses. As a result, it is evolutionarily conserved across different species [4–7]. Activation of the JAK-STAT pathway begins with the binding of an extracellular ligand to the cell surface receptor. This process triggers a cascade of complex steps, which includes the recruitment and subsequent phosphorylation of JAKs within the receptor complex, the phosphorylation and dimerization of STAT, the combination of STAT dimers with specific responsive element regions on the nucleus, and ultimately, the regulation of target gene transcription [8–11]. In this way, the extracellular signals and stimuli are relayed to the nucleus (Fig. 1). Under normal physiological conditions, the JAK-STAT signaling pathway regulates gene expression and cellular function by responding to extracellular signal molecules such as cytokines and growth factors. Numerous studies have demonstrated that JAK-STAT is involved in multiple biological processes, including cell proliferation, differentiation, apoptosis, immune response, hematopoietic regulation and embryonic development. Specifically, in the immune system, it participates in regulating the development, proliferation, and function of T cells and B cells; in the hematopoietic system, it controls the proliferation and differentiation of blood cells; and in embryonic development, it plays a role in organ formation and cell fate determination. Furthermore, the JAK-STAT signaling pathway can interact with other signaling pathways to form complex network regulatory systems. This network regulation helps maintain the biological balance of normal cells and ensures the normal function of tissues and organs.

The dysregulation of the JAK–STAT pathway has been found to contribute to carcinogenesis by promoting several oncogenic processes, including cell proliferation, invasion, metastasis, anti-apoptosis, immune escape, and angiogenesis [1, 12, 13]. Numerous studies have reported hyperactivation and frequent mutations in JAK–STAT signaling proteins in various human disorders, such as rheumatoid arthritis, inflammatory bowel disease, inflammatory skin conditions, myeloproliferative neoplasms, and solid tumors [14–17]. Considering its near-ubiquitous role in diverse diseases, an increasing number of small-molecule inhibitors or natural products targeting JAK-STAT proteins have been synthesized or developed [18–21]. Of them, many have been approved for clinical use and more selective inhibitors are currently undergoing clinical investigation [22–25].

Digestive tract tumors encompass a heterogeneous range of cancers, typically including esophageal, gastric, colorectal, liver, and pancreatic cancers [26, 27]. Despite the widespread availability of endoscopic examinations has greatly improved the early detection rates for certain digestive tract tumors, their non-specific symptoms and the limited therapeutic interventions for advanced-stage cancers pose a formidable challenge to improving the survival rates of patients [28–30]. Based on the GLOBOCAN 2020 statistics, digestive tract cancers comprised 23.4% of all cancer cases and 30.9% of all cancer-related deaths worldwide, emphasizing the substantial burden they impose on global public health [31, 32]. Hence, elucidating their pathogenesis and exploring novel biomarkers and therapeutic targets is imperative [33].

Advanced molecular biology and cutting-edge sequencing technologies have consistently identified the abnormal activation of JAK-STAT signaling in several digestive tract tumors. Moreover, the dysregulation of this pathway is associated with more malignant cell behaviors and tumor development, such as increased cell migration, invasion, and metastasis [34]. By regulating the reorganization of the cell skeleton and the expression of adhesion molecules, it promotes the migration ability of tumor cells [35–38]. At the same time, the JAK-STAT signaling pathway enhances the invasive ability of tumor cells by regulating the expression and activity of matrix metalloproteinases (MMPs), which can degrade extracellular matrix and provide invasion pathways for tumor cells [39–41]. In addition, it interacts with key transcription factors that promote epithelial-mesenchymal transition (EMT), making tumor cells more invasive [42–45]. These findings highlight that the JAK-STAT pathway significantly drives the progression and spread of digestive tract tumors. Reviewing its role in digestive tract tumors comprehensively will help us better understand its regulatory mechanisms and provide insights for developing more precise targeted treatment strategies.

Here, we have reviewed the involvement and mechanisms of the aberrantly activated JAK-STAT pathway in digestive tract tumors. Further, we have systematically analyzed the clinical significance of this pathway as a source of both potential biomarkers for early screening and therapeutic targets. Finally, we have discussed the application and prospects of targeting this pathway to enhance the clinical management of digestive tract tumors.

##  JAK-STAT pathway

The JAK-STAT pathway is a crucial signaling pathway inside the cell that involves two protein families: JAK and STAT [10].

The JAK family comprises four non-receptor tyrosine kinases, namely JAK1, JAK2, JAK3, and tyrosine kinase 2, which have a shared domain structure but distinct functions within the cell [34, 46, 47]. By analyzing, we have been able to understand the complete domain structure of JAKs with apparent molecular masses of 120–140 kDa [48]. JAKs are composed of seven Jak homology (JH) regions spanning four functional domains: a C-terminal tyrosine kinase domain formed by JH1, a pseudokinase domain constituted by JH2, a Src-homology 2 (SH2) domain comprising the JH3-JH4 regions, and an N-terminal FERM domain (band 4.1, ezrin, radixin, and moesin) containing the JH5-JH7 regions [49–51]. Thoroughly dissecting the functions and interactions of each domain within the JAK family will enhance our understanding of its role in signal transduction. The kinase domain, which is the most pivotal domain of JAKs, exhibits tyrosine kinase activity to phosphorylate target substrates like STATs, thus activating downstream signaling pathways and cellular responses [52]. Inhibiting the activity of the kinase domain usually disrupts an aberrantly activated JAK-STAT pathway, making this domain the major target for the development of JAK inhibitors [53–55]. The characteristic pseudokinase domain, which is beside the kinase domain, executes crucial regulatory functions rather than catalytic functions. It modulates JAK activation and substrate specificity by interacting with other proteins, but also prevents the excessive activation of JAK-STAT signaling by providing negative feedback [56]. Moreover, mutations in the pseudokinase domain have been proven to affect the basal activity of the kinase domain [57]. The SH2 domain functions as scaffolding to both facilitate the localization of JAK to activated receptors and phosphorylate STAT. As a result, it affects the nuclear translocation of STAT and downstream gene expression regulation [58–60]. The FERM domain is involved in interacting with transmembrane receptors and maintaining kinase activity [61, 62]. Several studies have reported that variations in the FERM domain contribute to aberrant JAK-STAT signaling in a wide range of human diseases [63, 64].

Signal transduction through the JAK-STAT pathway is mediated by four cytosolic JAKs situated near the cell membrane [65, 66]. Each JAK can bind to multiple types of cytokine receptors, resulting in different downstream effects [67]. JAK1, JAK2, and tyrosine kinase 2 are ubiquitously expressed in mammals, whereas the expression of JAK3 is predominantly restricted to hematopoietic, endothelial, and vascular smooth muscle cells [58, 68–71]. These four JAKs have been widely recognized as potential drug targets in diverse diseases, such as leukemia, polycythemia vera, myelofibrosis, essential thrombocythemia, cutaneous T-cell lymphoma, and inflammatory bowel disease [72–75]. However, the therapeutic effectiveness and safety of targeting the JAK family needs further clinical verification [76, 77].

The STAT family, first discovered while studying the activation of the interferon system in 1994, is a family of seven latent cytoplasmic transcription factors (STAT1-STAT4, STAT5A, STAT5B, and STAT6) in humans with a conserved separate window ranging from 750 to 850 amino acids [78–80]. Most STAT proteins possess similar structures: an N-terminal domain, a coiled-coil domain, a DNA-binding domain, a linker domain, an SH2 domain, and a C-terminal transactivation domain (STAT2 and STAT6 are exceptions because they lack the PMSP motif) [81–83]. The C-terminal transactivation domain is the main site for the phosphorylation of serine residues. The SH2 domain mediates STAT phosphorylation as well as the interaction between STATs and JAKs leading to STAT dimerization. The DNA-binding domain contains precise amino acid sequences to recognize and bind to specific DNA sequences, thus dictating DNA-binding specificity [84–88]. Multiple studies have discovered that different molecules activate specific STATs (especially STAT3 and STAT5) to initiate distinct regulatory mechanisms and functions that contribute to normal physiology as well as disease development [82, 89, 90]. Moreover, STAT3 and STAT5 are considered the most significant of all STATs because they are involved in malignant transformation [91–93]. Inhibiting constitutively activated STATs has also been demonstrated to suppress tumor growth, justifying the development of small-molecule STAT inhibitors to treat human cancers [94–96].

Excessive or prolonged activation of the JAK-STAT pathway is prevented by multiple molecules that form a negative feedback loop to regulate the duration and intensity of the pathway. Activated STATs stimulate suppressor of cytokine signaling (SOCS) proteins, which inhibit the further activation of STAT signaling by competing with STATs for binding, ubiquitinating and degrading SOCS substrates, and directly repressing JAK activity [97–99]. Protein inhibitors of activated STATs (PIAS) also negatively regulate JAK-STAT signaling by blocking the STAT-DNA interaction, inducing protein SUMOylation, and recruiting transcriptional co-repressors to STAT target genes [100, 101]. In addition, protein tyrosine phosphatases dephosphorylate activated STATs, leading to the inactivation and termination of STAT signaling [102, 103].

## Abnormal JAK-STAT pathway in digestive tract tumors

**Fig. 2:**
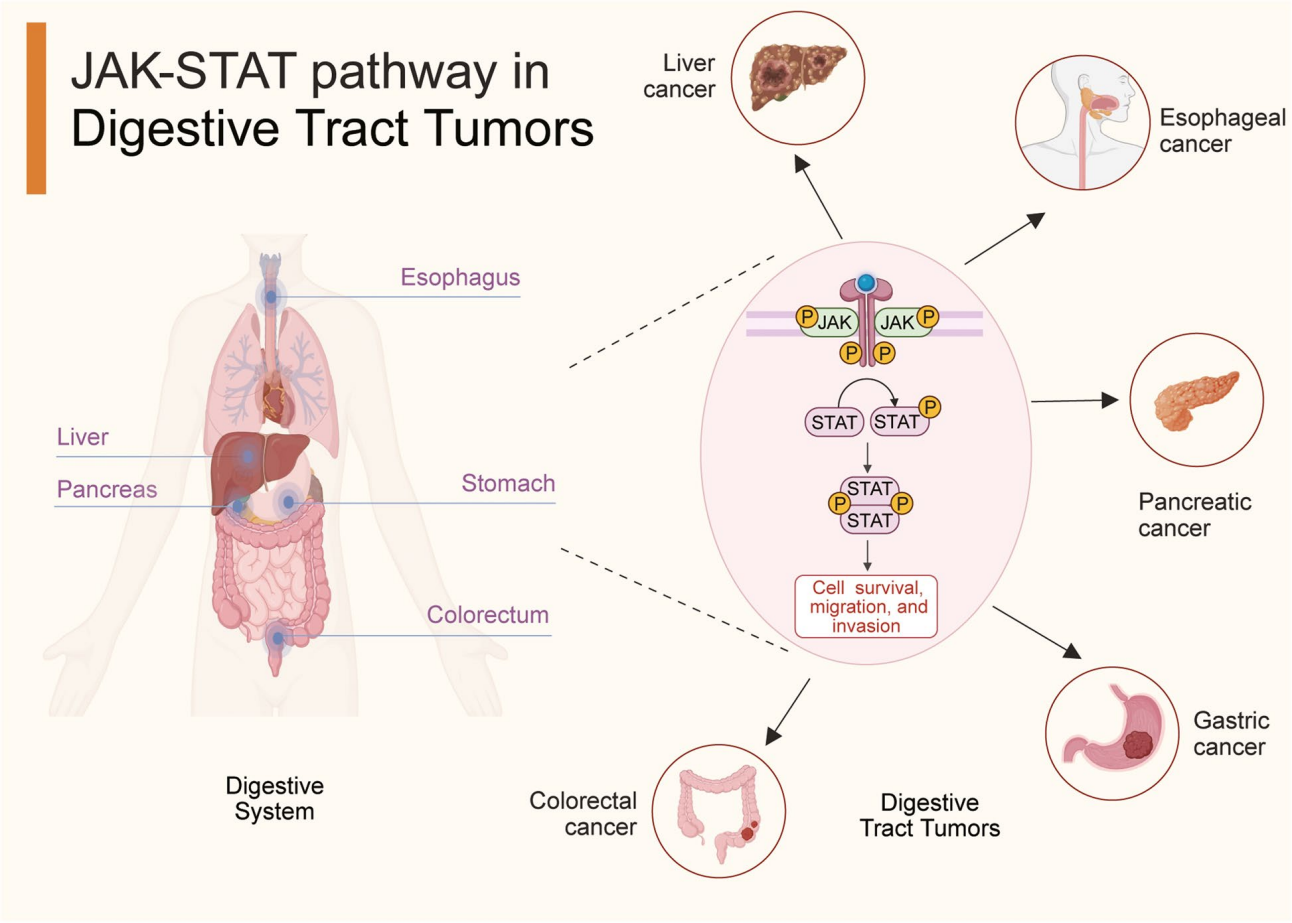
The involvement of the JAK-STAT pathway in digestive tract tumors

The JAK pathway is aberrantly activated in multiple digestive tract tumors due to mutations in JAKs or STATs, gene fusions with JAKs or STATs, and the restrained expression of negative regulators, ultimately engendering tumor cell malignant behaviors, such as proliferation, invasion, drug resistance, immune escape, and metastasis [3, 104–107]. A coherent understanding of the aberrant activity of this pathway can help researchers devise therapeutic strategies to decelerate tumor progression [108]. The JAK-STAT signaling pathway has emerged as a potential therapeutic target in gastrointestinal tumor treatment [109–111]. One intriguing aspect is the involvement of JAK-STAT signaling in chemotherapy resistance in digestive tract tumors [112, 113]. Emerging evidence underscores the pivotal role of aberrant JAK-STAT pathway activation in conferring resistance to commonly used chemotherapy agents in the clinical management of digestive tract tumors [114–117]. This aberrant activation has been linked to the upregulation of anti-apoptotic proteins in tumor cells, thereby imparting resistance to chemotherapy-induced apoptosis in digestive tract tumors [118, 119]. Additionally, within the realm of treatment resistance, cancer stem cells (CSCs) have garnered significant attention due to their involvement in tumorigenesis, metastasis, and therapy resistance [120–122]. Studies have elucidated that JAK-STAT signaling fosters the stemness properties of CSCs in digestive tract tumors, ultimately contributing to therapy resistance and tumor recurrence [45, 123, 124]. The compelling body of research pointing to the involvement of JAK-STAT signaling in chemotherapy resistance underscores the potential of targeting this pathway as a promising strategy for surmounting treatment obstacles in digestive tract tumors. A comprehensive understanding of the intricate interplay between JAK-STAT signaling, tumor cell apoptosis, and the stemness properties of CSCs will be instrumental in shaping effective therapeutic interventions to combat chemotherapy resistance and improve patient outcomes in the clinical management of digestive tract tumors. Currently, several JAK-STAT inhibitors are available for clinical use in diverse diseases, such as rheumatoid arthritis, myeloproliferative neoplasms, and inflammatory bowel disease [9, 19, 23]. The efficacy of some inhibitors, particularly of digestive tract tumors, is currently being optimized, and combination therapies are being explored to achieve better clinical outcomes [13, 18, 125, 126]. The heterogeneity of different malignant digestive tract tumors and individual variability warrant further in-depth research to determine the efficacy and safety of a particular treatment strategy [127]. Here, we have summarized the carcinogenic mechanisms of dysregulated JAK-STAT signaling in digestive tract tumors, including esophageal, gastric, colorectal, liver, and pancreatic cancer (Fig. 2). We have also presented the efficacy and mechanism of some JAK-STAT inhibitors used for managing digestive tract tumors.

### Liver cancer

**Fig. 3:**
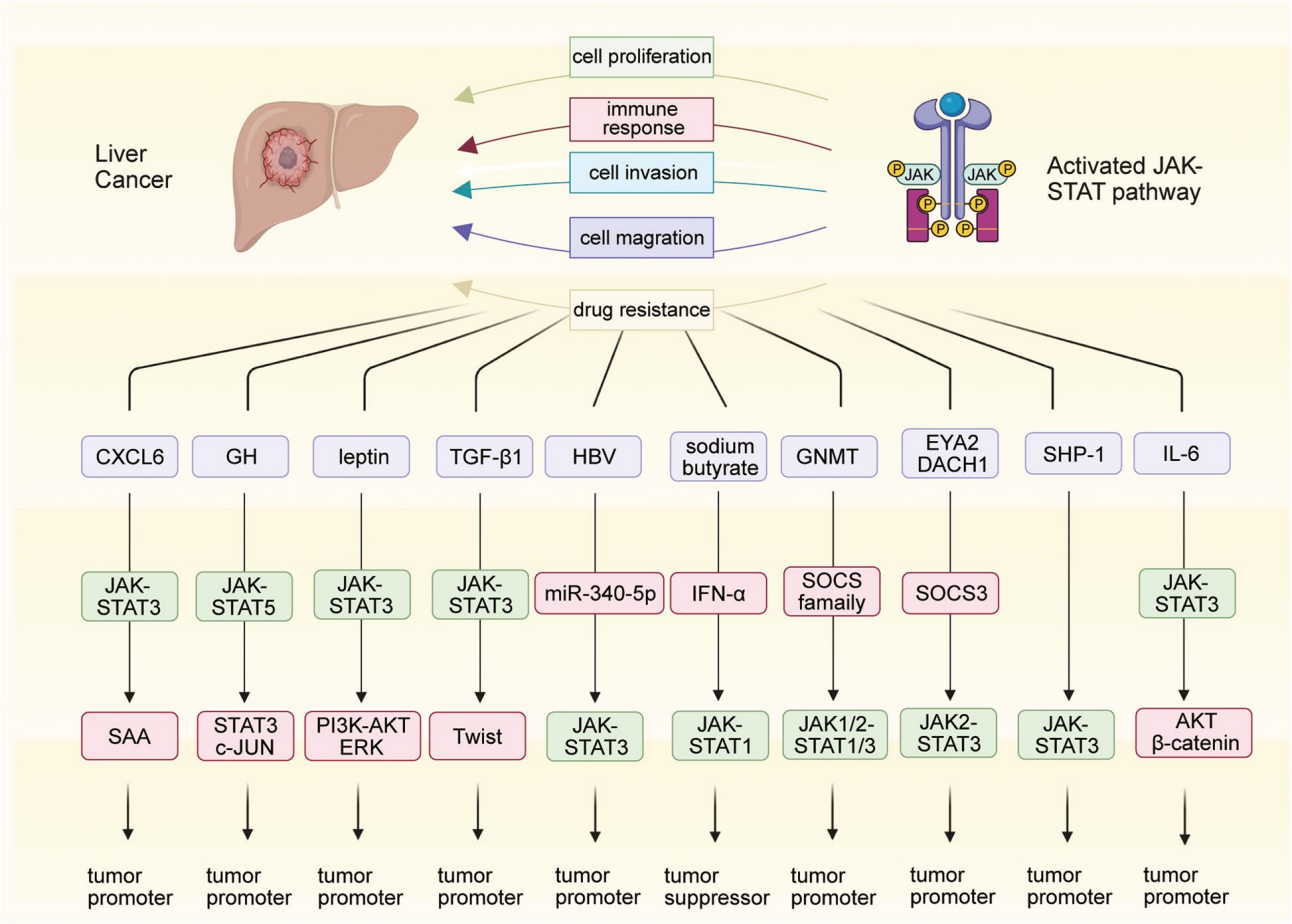
The regulatory mechanisms of the JAK-STAT pathway in the progression of liver cancer

Numerous studies have established that the ubiquitous activation and mutations to the JAK-STAT pathway are essential determinants of tumor development and drug sensitivity in liver cancer (Fig. 3) [128, 129]. Recently, tumor margins have received considerable attention. These regions are known to significantly influence the infiltration and invasion of tumor cells [130–133]. A detailed assessment of the characteristics and biological properties of tumor margins offers better insights into the development of anti-angiogenesis therapies, tumor invasiveness, and the risk of recurrence [134, 135]. A spatial transcriptomic analysis of liver cancer revealed that JAK-STAT3 signaling, abnormally activated by C-X-C motif chemokine ligand 6, induced the damaged hepatocytes in tumor margins to highly express serum amyloids A1 and A2. This led to macrophage accumulation and M2 polarization, facilitating local immunosuppression and liver cancer progression [136, 137].

Growth hormone (GH) crucially regulates human longitudinal growth, metabolism, and tissue repair by directly or indirectly acting on the liver [138, 139]. Tumor-derived GH has been comprehensively linked with the pathogenesis and progression of various cancers, such as liver, breast, and prostate cancers [140–143]. Sustained exposure to high levels of GH can cause liver cancer to occur frequently and develop aggressively [144–148]. The loss of STAT5 in liver cells reverses the pathological changes associated with chronic inflammation caused by the overactivation of GH signaling; however, it leads to the earlier occurrence of liver cancer with a more aggressive phenotype. The loss of STAT5 is compensated by the activation of the STAT3 and c-JUN pathways to facilitate the malignant transformation of hepatocytes. This may be attributed to the synthetic actions of lipodystrophy, the deletion of hepatic protective mediators, the activation of the STAT3-c-JUN pathways, and DNA damage [149].

Leptin is a peptide hormone that plays an important role in broad biological processes, including energy metabolism, appetite regulation, and insulin sensitivity [150, 151]. Emerging studies have suggested that abnormalities in leptin levels are correlated with the carcinogenic processes of diverse cancers [152–155]. Leptin was found to enhance the malignant properties, such as cell invasion and migration potential, of both HepG2 and Huh7 cells by stimulating the JAK-STAT-phosphoinositide 3-kinase-AKT-extracellular signal-regulated kinase (ERK) axis. In the absence of the STAT3 inhibitor AG490, leptin-induced malignant behaviors were notably restrained, further confirming the powerful carcinogenic effect of leptin in liver cancer [156]. Transforming growth factor β1 markedly induced the migration and invasion of liver cancer cells by promoting epithelial-to-mesenchymal transition. It activated JAK-STAT3 signaling and further upregulated Twist in HepG2 cells, whose enhanced migratory and invasive abilities were reversed after AG490 treatment [157].

Hepatitis B virus (HBV) was also found to contribute to cell migration in liver cancer. In vitro experiments showed that HBV rescued the inhibition of cell migration by downregulating miR-340-5p and elevating STAT3 levels [158]. Interferon-alpha (IFN-α) is a well-known treatment option for HBV-induced hepatitis [159–161]. Recent studies have reported that sodium butyrate, a differentiation inducer, arrested cell proliferation and strengthened the anti-tumor efficacy of IFN-α in liver cancer by specifically activating STAT1 and enhancing IFN-α-mediated STAT1 expression [162].

The glycine N-methyltransferase (*GNMT*) gene functions as a tumor susceptibility gene for liver cancer and exhibits a unique tissue expression pattern [163–165]. *GNMT*, which is generally expressed in normal liver tissue, is found to be undetectable in liver cancer and shows attenuated expression in the livers of patients at risk of developing hepatocellular carcinoma [166, 167]. Knocking out *GNMT* in mice liver activated JAK-STAT pathways, which promoted the malignant transformation of normal liver cells, accompanied by the downregulation of SOCS1, SOCS2, SOCS3, and cytokine-inducible SH2-containing protein and the upregulation of JAK1/2, STAT1, and STAT3 [168].

Eyes absent homolog 2 (*EYA2*) is considered a tumor suppressor gene in liver cancer, usually exhibiting a pattern of somatic mutations (p.Ala510Glu). Downregulated *EYA2* was found to transcriptionally upregulate *SOCS3* with the help of dachshund homolog 1. SOCS3 further blocked the JAK2-STAT3 pathway to check the progression of liver cancer [169]. In addition, SH2 domain-containing phosphatase 1, a tumor suppressor of liver cancer, was detected to be markedly downregulated in human liver cancer tissues and associated with poor overall survival. Knocking it down enhanced the activity of the JAK-STAT3 pathway to aggravate hepatocarcinogenesis and exacerbate the malignant phenotype of liver cancer [170]. Akt/β-catenin-driven tumors possess a subtype of side population/CD44 + tumorigenic cells with stem/progenitor-like properties that develop resistance to chemotherapeutic drugs. Targeting the JAK-STAT3 pathway has shown great promise in patients with Akt/β-catenin-driven liver cancer [171].

### Gastric cancer

Numerous studies have reported the constitutive activation of STAT3 in gastric cancers as well as its tight association with the prognosis and clinicopathological characteristics of gastric cancer patients [172–174]. STAT3 is known to exert its oncogenic effects and regulate various malignant cell behaviors in gastric cancer by interacting with diverse downstream targets [175, 176]. A previous study demonstrated that STAT3 directly upregulated Toll-like receptor 2, an inflammatory mediator, to inhibit epithelial proliferation and anti-apoptosis, thereby enhancing tumorigenesis instead of inflammation in gastric cancer [177]. The integrity of the gastric mucosa is protected by trefoil factor 1 (TFF1), a small cysteine-rich acidic secreted protein that exerts both anti-inflammatory and pro-apoptotic effects [178–180]. Recently, a study showed that the loss of TFF1 is responsible for the activation of STAT3. TFF1 impeded the combination of interleukin-6 (IL-6) with IL-6 Rα and further disrupted the activation of STAT3 in the gastric cancer cell lines AGS and STKM2 [181].

Tumor-associated macrophages constitute a large proportion of the infiltrating inflammatory cells in the tumor microenvironment (TME) and display remarkable versatility and plasticity [182–184]. Recently, macrophages were identified to secrete CXCL8 under hypoxic conditions, which hyperactivated the JAK-STAT1 pathway in gastric cancer by interacting with C-X-C motif chemokine receptor 1/2 (CXCR1/2) on the cell membrane. Subsequently, IL-10 was overexpressed and M2-type macrophages became polarized, establishing a positive feedback loop between macrophages and gastric cancer progression [185]. Tumor-associated macrophages have also been shown to be tightly associated with stimulator of interferon genes (STING), which is indispensable for regulating the innate and adaptive immune systems. Knocking-down this regulator induced macrophages to differentiate into pro-inflammatory subtypes via the IL-6R-JAK-STAT-IL-24 pathway, thus achieving pro-apoptotic effects in gastric cancer [186].

Dendritic cells function as antigen-presenting cells to dynamically balance the immune response [187–189]. Recent flow cytometry results suggest that *YTHDF1* knockout recruited dendritic cells and consequently, enhanced the infiltration of T helper cells and cytotoxic T cells in the TME of gastric cancer, promoting the reactivation of adaptive antitumor immunity. *YTHDF1* knockout was found to upregulate type I IFN-γ and trigger the JAK-STAT1 pathway to maintain a sustainable systemic antitumor immunity [190].

Additionally, emerging studies have indicated that tumor-infiltrating neutrophils account for the most influential components in the gastric cancer TME and are correlated with poor patient survival [191–193]. A novel FasL^+^PD‐L2^+^ neutrophil phenotype was discovered in advanced gastric cancer, and these cells exerted immunosuppressive effects in tumor development. Mechanistically, T helper 17 cells secrete IL‐17 A, which subsequently triggers the ERK-nuclear factor κB (NF‐κB) pathway and contributes to the expression of FasL on neutrophils. Tumor‐derived granulocyte colony-stimulating factor markedly activated the JAK-STAT3 pathway and further upregulated programmed cell death-ligand 2 (PD-L2) in neutrophils [194]. Tumor-activated neutrophils also highly expressed PD-L1 and strongly retarded the immunity of normal T cells. Granulocyte macrophage colony-stimulating factor in the TME activated the JAK-STAT3 pathway to upregulate PD-L1 in neutrophils, thus promoting tumor-related immunosuppression and progression [195]. In addition, NF-κB1 polymorphisms were found associated with pro-tumorigenic activity in diverse human cancers, especially digestive tract tumors [196–198]. The loss of NF-κB1 in the gastric epithelial and hematopoietic compartments resulted in abnormal gastric inflammation and invasive tumor progression [199]. It also contributed to the overexpression of tumor necrosis factor and STAT1 to further increase the expression of inflammatory effectors and inhibitory immune checkpoint regulators, thereby exacerbating inflammation-associated tumor development [200, 201].

### Colorectal cancer

Colorectal cancer is a multifarious disease that involves the dysregulation of multiple signaling pathways, including the JAK-STAT pathway, which regulates the tumor growth, proliferation, migration, and self-renewal characteristics [202–204]. Notably, a conspicuous local inflammatory reaction is correlated with improved survival of colorectal cancer patients, whereas an elevated systemic inflammatory response is correlated with worse clinical outcomes [205–208]. STAT3 levels were found to be especially elevated in stage I-III colorectal cancer patients undergoing surgery, leading to abnormal local and systemic inflammatory responses and poorer prognoses [209]. Numerous studies have shown that the constitutive activation of STAT3 in colorectal cancer drives cell proliferation and tumor growth, thus providing novel insights into treating this disease [210].

Using a transgenic mouse model (∆133p53 isoform) prone to tumors, researchers showed that IL-6 drove the oncogenic activity of the ∆133p53 isoform by upregulating the JAK-STAT3 pathway. Moreover, overexpression of ∆133TP53 mRNA in human colorectal cancers signified a more aggressive tumor phenotype and poorer patient prognosis [211]. The protein tyrosine kinases BMX and HCK were shown to significantly activate the JAK-STAT3 pathway, which promoted the hyperproliferative characteristics of normal epithelial NCM460 cells and initiated adenoma formation in human intestinal organoids. These results contribute to our understanding of adenoma-carcinoma transformation during colorectal carcinogenesis [212].

The circular RNA circSPARC was found upregulated in colorectal cancer, where it served as a competing endogenous RNA to combine with miR-485-3p, thus elevating JAK levels, STAT3 phosphorylation, and STAT3 nuclear translocation. These changes ultimately accelerated tumor growth and metastasis of colorectal cancer [36]. In addition, the long non-coding RNA FEZF1-AS1 was discovered to be overexpressed in colorectal cancer tissues and was associated with poor patient survival. Functional analysis revealed that FEZF1-AS1 upregulated pyruvate kinase 2 to promote aerobic glycolysis and further activate STAT3 signaling. These FEZF1-AS1-induced changes accelerated cell proliferation and metastasis in colorectal cancer [213].

Studies have also explored the regulatory relationship between STAT3 and microRNAs, which ultimately influences tumor oncogenesis [214–216]. Elevated miR-572 expression and downregulation of modulator of apoptosis-1 were observed in colorectal cancer with high expression of STAT3. Mechanistically, STAT3 increased miR-572 levels to inhibit the expression of modulator of apoptosis-1, leading to enhanced cell growth, migration, and invasion in colorectal cancer [217]. In addition, PIAS3, a negative regulator of STAT signaling, was found to decrease the expression of miR-18a to restrain the activity of NF-κB and STAT3 in an azoxymethane-dextran sulfate sodium-induced mouse model. The PIAS3-mediated feedback loops exhibited the powerful ability to control cell proliferation in the progression of colitis-associated colorectal cancer, thus offering promising therapeutic targets [218].

### Pancreatic cancer

Aberrant stimulation of JAK-STAT signaling also contributes to the oncogenesis of pancreatic cancer [219, 220]. Patients with high STAT3 expression exhibited advanced tumor clinicopathological parameters and worse survival [219]. IL-6 activated STAT3 and increased its phosphorylation, thus upregulating matrix metalloproteinase 2 and vascular endothelial growth factor in the pancreatic cancer line Capan-2. The STAT3-mediated enhanced invasion of Capan-2 cells was counteracted by AG490 [221].

Stellate cells in the TME of pancreatic cancer also secrete IL-6 and drive the activation of the JAK2-STAT3 pathway, which leads to the accumulation of myeloid-derived suppressor cells and the maintenance of an immunosuppressive microenvironment [222]. TEA domain transcription factor 2 was found to upregulate CD109 in the basal-like subtype cells of pancreatic cancer, subsequently hyperactivating the JAK-STAT3 pathway and leading to enhanced metastasis [223]. Pancreatitis was demonstrated to mediate acinar-to-ductal metaplasia and gradually evolve into pancreatic cancer [224].

Numerous studies have validated that the *KRAS* oncogene is commonly mutated in the early stages of pancreatic cancer [225, 226]. *KRAS* mutations were found to upregulate the transcriptional regulators yes-associated protein 1 and transcriptional coactivator with PDZ-binding motif to further activate the JAK-STAT3 pathway, thus reprogramming acinar cells and initiating tumorigenesis [227]. Moreover, elevated IL-22 levels during pancreatic tumor development affected the plasticity of acinar cells and induced ductal formation, epithelial-to-mesenchymal transition, and tumor metastasis, all of which were reversed by inhibiting the JAK-STAT3 pathway [228].

IFN-α induced the survival response of human epidermoid cancer cells by hyperactivating the RAS-RAF1-MEK1-ERK1/2 pathway in an epidermal growth factor (EGF)-dependent manner [229, 230]. The activation of peroxisome proliferator-activated receptor γ also enhanced pancreatic cancer cell invasion and migration through diverse mechanisms involving crosstalk with STAT3 [231–234]. Given these findings, researchers explored the synergistic effect of IFN-β and troglitazone, an agonist of peroxisome proliferator-activated receptor γ, on the growth and autophagy of the pancreatic cancer cell line BxPC-3. IFN-β and troglitazone together exerted a stronger inhibitory influence on STAT3-dependent escape pathways involving the activation of STAT3, mitogen-activated protein kinase, and AKT [235].

Pancreatic cancer patients displayed increased levels of prolactin (PRL). PRL induced the phosphorylation of the JAK2-STA3-ERK-AKT pathway to facilitate the formation of pancospheres and enhance the migratory capacity of cells. These pro-cancer effects of PRL were counteracted by some antipsychotic drugs like penfluridol in pancreatic cancer mouse models [236].

### Esophageal cancer

STAT3 plays pivotal roles in esophageal cancer as well. Activated STAT3 acts as an oncogene in esophageal cancer by promoting cell viability, tumor angiogenesis, and metastasis [110, 237–239]. Polo-like kinase 1 (PLK1) is preclinically considered a functional regulator in multiple critical cell events during tumor progression [240–242]. PLK1 was found overexpressed in esophageal cancer and showed promising prognostic efficacy [243]. Constitutively activated STAT3 and positively regulated PLK1 collectively enhanced proliferation and apoptosis resistance in the esophageal cancer cell line KYSE510 [244].

Similarly, EGF receptor was also shown to augment cell migration in esophageal cancer. The EGF receptor-mediated phosphorylation of STAT1 at Tyr701 led to the formation of the STAT1-STAT3 complex and its translocation into the nucleus. JAK-STAT signaling also upregulated matrix metalloproteinase-1, thereby increasing keratinocyte migration in esophageal cancer [245].

Furthermore, elevated levels of ring finger protein 168 were reported to contribute to malignant cell proliferation and invasion in esophageal cancer. Ring finger protein 168 repressed STAT1 polyubiquitination and degradation to upregulate JAK-STAT1 signaling and the downstream functional genes, contributing to the growth and invasion of esophageal cancer [246]. Small nucleolar RNA host gene 6 was discovered to be markedly upregulated in KYSE150 and KYSE450 cells, and was positively associated with colony formation, migration, tumor malignancy, and 5-fluorouracil resistance in esophageal cancer. It increased the levels of enhancer of zeste homolog 2 to promote STAT3 phosphorylation and H3K27me3 expression, thereby enhancing 5-fluorouracil resistance [247].

Additionally, JAK-STAT signaling pathway plays a crucial role not only through its direct influence on tumor cell survival, proliferation, and therapy resistance, but also through the crosstalk with other signaling pathways [248–250]. The interaction between JAK-STAT signaling and other pathways is essential for the regulation of tumor development and progression in digestive tract tumors [251–254]. One vital cross-interaction in digestive tract tumors involves the interplay between JAK-STAT and PI3K-AKT signaling pathways [255, 256]. The crosstalk between JAK-STAT and PI3K-AKT pathways creates a positive feedback loop that amplifies tumor-promoting signals and contributes to tumor growth and therapeutic resistance in digestive tract tumors [257, 258]. In addition to the PI3K-AKT pathway, JAK-STAT signaling also interacts with other major signaling pathways, such as the MAPK-ERK pathway, contributing to the aggressive phenotype and therapy resistance observed in digestive tract tumors [259]. The cross-talk between JAK-STAT signaling and other pathways highlights the importance of a comprehensive understanding of the intricate network of molecular interactions in cancer progression and therapy response of digestive tract tumors. Targeting multiple signaling pathways simultaneously may be a promising approach to overcome therapy resistance and improve patient outcomes in digestive tract tumors.

## Conclusion and prospects

In conclusion, the JAK-STAT pathway has emerged as a crucial factor in the pathogenesis of digestive tract tumors. Its aberrant activation, triggered by pro-inflammatory cytokines, disrupts various biological processes such as cell growth, apoptosis, and migration. The current understanding of the classic activation and regulation of the JAK-STAT pathway has provided a foundation for identifying potential therapeutic targets for digestive tract tumors. However, recent research has shown that the use of JAK inhibitors raises safety concerns due to their lack of specificity, as they inhibit multiple signal transduction pathways. Therefore, careful monitoring and management of infection complications is imperative when administering JAK inhibitors to treat digestive tract tumors.

To fully understand the mechanisms underlying the JAK-STAT pathway in digestive tract tumors, future research should focus on gaining a better understanding of the interplay between this pathway and other signaling pathways. Additionally, it is important to identify specific molecular targets within the JAK-STAT pathway that can be selectively modulated to achieve maximal therapeutic benefit. Recent preclinical and clinical trials have shown promising results with drugs targeting this pathway; however, striking a balance between efficacy and safety remains a challenge.

In summary, a better understanding of the JAK-STAT pathway in digestive tract tumors will pave the way for the development of targeted therapies that are both safe and effective. Further research is needed to fully elucidate the mechanisms involved in the dysregulation of this pathway and to optimize therapeutic strategies for the treatment of digestive tract tumors Tables 1 and 2.

**Table 1:** Expression and outcomes of the JAK–STAT pathway in digestive tract tumors

Cancer	Expression in cancers	Outcomes of the activated JAK-STAT pathway in cancers	Year	Refs
Liver cancer	upregulation of JAK1, JAK2, JAK3, and STAT3	enhanced immunosuppression, and tumor metastasis	2023	[136]
Liver cancer	upregulation of JAK2, and STAT3	enhanced cell proliferation, clone formation, invasion, and migration	2021	[169]
Liver cancer	upregulation of JAK1, JAK2, TYK2, STAT1, STAT3, and STAT5	enhanced cell anti-apoptosis	2006	[129]
Liver cancer	upregulation of JAK1, JAK2, STAT1, and STAT3	enhanced cell proliferation	2008	[168]
Liver cancer	upregulation of STAT3	enhanced cell migration, and invasion	2018	[157]
Liver cancer	upregulation of STAT3	enhanced cell proliferation	2007	[156]
Liver cancer	upregulation of STAT3	enhanced cell migration	2017	[158]
Liver cancer	upregulation of STAT3	enhanced cell proliferation, migration, invasion, and tumorigenicity	2018	[170]
Liver cancer	upregulation of STAT3	enhanced tumor formation, and drug resistance	2020	[171]
liver cancer	downregulation of STAT1	enhanced cell growth arrest, and the responsiveness to IFN-α	2018	[162]
Liver cancer	downregulation of STAT5	enhanced hepatoprotective functions	2012	[149]
Gastric cancer	upregulation of JAK1, JAK2, and STAT1	enhanced cell proliferation and repression	2022	[190]
Gastric cancer	upregulation of STAT3	enhanced cell proliferation and anti-apoptosis	2012	[177]
Gastric cancer	upregulation of STAT3	enhanced dysplastic lesions and loss of mucosal integrity	2019	[181]
Gastric cancer	upregulation of STAT3	enhanced the immunosuppression of FasL + PD-L2 + neutrophils, and tumor growth	2022	[194]
Gastric cancer	upregulation of STAT3	enhanced the immunosuppression of PD-L1 + neutrophils, and tumor growth	2017	[195]
Gastric cancer	upregulation of STAT1	enhanced inflammatory immune response	2020	[201]
Gastric cancer	upregulation of STAT1	enhanced the polarization of M2-type macrophage	2022	[185]
Gastric cancer	upregulation of STAT1, and STAT3	enhanced inflammation, and immune evasion	2018	[200]
Gastric cancer	upregulation of STAT1, and STAT3	enhanced immunosuppression, and anti-apoptosis	2020	[186]
Colorectal cancer	upregulation of JAK2, and STAT3	enhanced cell migration and proliferation	2021	[36]
Colorectal cancer	upregulation of STAT3	enhanced cell proliferation, and adenoma formation	2022	[212]
Colorectal cancer	upregulation of STAT3	enhanced cell invasion	2018	[211]
Colorectal cancer	upregulation of STAT3	enhanced cell proliferation, and metastasis	2018	[213]
Colorectal cancer	upregulation of STAT3	enhanced cell growth, migration, and invasion	2018	[217]
Colorectal cancer	upregulation of STAT3	enhanced cell proliferation	2018	[218]
Pancreatic cancer	upregulation of STAT3	enhanced chemotherapy resistance	2023	[223]
Pancreatic cancer	upregulation of STAT3	enhanced acinar-to-ductal metaplasia	2016	[227]
Pancreatic cancer	upregulation of STAT3	enhanced acinar to ductal metaplasia, stem cell features, and the epithelial-mesenchymal transition	2020	[228]
Pancreatic cancer	upregulation of STAT3	enhanced tumor growth inhibition, and inhibited autophagic death	2012	[235]
Esophageal cancer	upregulation of JAK1, JAK2, STAT1, and STAT3	enhanced cell migration	2004	[245]
Esophageal cancer	upregulation of STAT3	enhanced tumor survival, and proliferation	2012	[244]
Esophageal cancer	upregulation of STAT3	enhanced 5-FU resistance	2023	[247]
Esophageal cancer	upregulation of STAT1	enhanced tumor growth, and invasion	2019	[246]

**Table 2:** The roles and mechanisms of the JAK–STAT pathway in digestive tract tumors

Human Diseases	Regulatory Mechanism of JAK-STAT pathway	Roles of the activated JAK-STAT pathway in cancers	Refs
Liver cancer	CXCL6, JAK-STAT3 pathway, and SAA	tumor promoter	[136]
Liver cancer	EYA2, DACH1, SOCS3, JAK2-STAT3 pathway	tumor promoter	[169]
Liver cancer	GH, and STAT5	tumor suppressor	[149]
Liver cancer	GNMT, and JAK-STAT3 pathway	tumor promoter	[168]
Liver cancer	TGFβ1, JAK-STAT3 pathway, and Twist	tumor promoter	[157]
Liver cancer	leptin, JAK-STAT3 pathway, PI3K-AKT pathway, ERK signaling	tumor promoter	[156]
Liver cancer	Hepatitis B virus, miR-340-5p, and JAK-STAT3 pathway	tumor promoter	[158]
Liver cancer	sodium butyrate, JAK-STAT1 pathway, and IFN-α	tumor suppressor	[162]
Liver cancer	SHP-1, and JAK-STAT3 pathway	tumor promoter	[170]
Liver cancer	JAK-STAT3 pathway, AKT pathway, and β-catenin pathway	tumor promoter	[171]
Gastric cancer	JAK-STAT3 pathway, and TLR2	tumor promoter	[177]
Gastric cancer	TFF1, and IL6-JAK-STAT3 pathway	tumor promoter	[181]
Gastric cancer	G-CSF, JAK‐STAT3 pathway, and PD‐L2	tumor promoter	[194]
Gastric cancer	GM-CSF, JAK-STAT3 pathway, and PD-L1	tumor promoter	[195]
Gastric cancer	NF-κB1, JAK-STAT1 pathway, and PD-L1	tumor promoter	[200]
Gastric cancer	NF-κB1, JAK-STAT1 pathway, TNF, and PD-L1	tumor promoter	[201]
Gastric cancer	CXCL8, CXCR1/2, JAK-STAT1 pathway, and IL-10	tumor promoter	[185]
Gastric cancer	YTHDF1, IFNGR1, and JAK1/2-STAT1 pathway	tumor promoter	[190]
Gastric cancer	STING, IL-6R-JAK-STAT1 pathway, and IL-24	tumor promoter	[186]
Colorectal cancer	BMX, HCK, and the JAK-STAT3 pathway	tumor promoter	[212]
Colorectal cancer	circSPARC, miR-485-3p, and JAK2-STAT3 pathway	tumor promoter	[36]
Colorectal cancer	IL-6-JAK-STAT3 pathway	tumor promoter	[211]
Colorectal cancer	lncRNA FEZF1-AS1, PKM2, and JAK-TAT3 pathway	tumor promoter	[213]
Colorectal cancer	JAK-STAT3 pathway, miR-572, MOAP-1	tumor promoter	[217]
Colorectal cancer	NF-κB, JAK-STAT3 pathway, miR-18a, and PIAS3	tumor promoter	[218]
Pancreatic cancer	TEAD2, CD109, and JAK-STAT3 pathway	tumor promoter	[223]
Pancreatic cancer	KRAS, JAK-STAT3 pathway, YAP1, and TAZ	tumor promoter	[227]
Pancreatic cancer	IL-22, JAK-STAT3 pathway, and TWIST	tumor promoter	[228]
Pancreatic cancer	IFN-β, PPAR-γ, and JAK-STAT3 pathway	tumor promoter	[235]
Esophageal cancer	JAK-STAT3 pathway, and PLK1	tumor promoter	[244]
Esophageal cancer	RNF168, and JAK-STAT1 pathway	tumor promoter	[246]
Esophageal cancer	SNHG6, JAK-STAT3 pathway, and EZH2	tumor promoter	[247]
Esophageal cancer	EGFR, JAK1/2-STAT1/3 pathway, and MMP-1	tumor promoter	[245]

## Data Availability

Not applicable.
